# Prognostic Values of Baseline ^18^F-FDG PET/CT in Patients with Peripheral T-Cell Lymphoma

**DOI:** 10.1155/2020/9746716

**Published:** 2020-02-25

**Authors:** Yeye Zhou, Xiaoyi Zhang, Haifeng Qin, Zixuan Zhao, Jihui Li, Bin Zhang, Shibiao Sang, Yiwei Wu, Shengming Deng

**Affiliations:** ^1^Department of Nuclear Medicine, The First Affiliated Hospital of Soochow University, Suzhou, China; ^2^Department of Nuclear Medicine, Changshu No. 2 People's Hospital, Changshu, China; ^3^Department of Nuclear Medicine, First People's Hospital of Kunshan, Kunshan, China

## Abstract

**Purpose:**

In the present study, we aimed to investigate whether the metabolic parameters on baseline ^18^F-fluorodeoxyglucose positron emission tomography/computed tomography (^18^F-FDG PET/CT) could be used to predict prognosis in peripheral T-cell lymphomas (PTCL).

**Methods:**

A total of 51 nodal PTCL patients who underwent baseline ^18^F-FDG PET/CT were retrospectively evaluated in the present study. Total metabolic tumor volume (TMTV), total lesion glycolysis (TLG), and maximum standardized uptake value (SUV_max_) were also assessed. Besides, the National Comprehensive Cancer Network International Prognostic Index (NCCN-IPI) was also included. Log-rank test and Cox regression analysis were used to evaluate progression-free survival (PFS) and overall survival (OS).

**Results:**

The median follow-up was 18 months. Patients with low TLG, TMTV, and SUV_max_ levels had a significantly better clinical outcome than those with high TLG, TMTV, and SUV_max_ levels. The 2-year PFS rates of the high- and low-TMTV groups were 34.62% and 80%, respectively (*p* < 0.001), whereas the corresponding 2-year OS rates were 46.15% and 84.00%, respectively (*p* < 0.001), whereas the corresponding 2-year OS rates were 46.15% and 84.00%, respectively (*p* < 0.001), whereas the corresponding 2-year OS rates were 46.15% and 84.00%, respectively (*p* < 0.001), whereas the corresponding 2-year OS rates were 46.15% and 84.00%, respectively (*p* < 0.001), whereas the corresponding 2-year OS rates were 46.15% and 84.00%, respectively (*p* < 0.001), whereas the corresponding 2-year OS rates were 46.15% and 84.00%, respectively (*p* < 0.001), whereas the corresponding 2-year OS rates were 46.15% and 84.00%, respectively (*p* < 0.001), whereas the corresponding 2-year OS rates were 46.15% and 84.00%, respectively (*p* < 0.001), whereas the corresponding 2-year OS rates were 46.15% and 84.00%, respectively (*p* < 0.001), whereas the corresponding 2-year OS rates were 46.15% and 84.00%, respectively (*p* < 0.001), whereas the corresponding 2-year OS rates were 46.15% and 84.00%, respectively (*p* < 0.001), whereas the corresponding 2-year OS rates were 46.15% and 84.00%, respectively (*n* = 10), intermediate-risk group with TMTV > 62.405 or NCCN-IPI score of 4-8 (2-year PFS and OS were 52.4% and 66.7%, respectively, *n* = 10), intermediate-risk group with TMTV > 62.405 or NCCN-IPI score of 4-8 (2-year PFS and OS were 52.4% and 66.7%, respectively, *n* = 10), intermediate-risk group with TMTV > 62.405 or NCCN-IPI score of 4-8 (2-year PFS and OS were 52.4% and 66.7%, respectively,

**Conclusions:**

Baseline TMTV and TLG were independent predictors of PFS and OS in PTCL patients, and SUV_max_ and NCCN-IPI scores were also independent predictors of OS. Moreover, the combination of TMTV and NCCN-IPI scores improved patient risk-stratification at the initial stage and might contribute to the adjustment of the therapeutic regime. This trial is registered with ChiCTR1900025526.

## 1. Introduction

Peripheral T-cell lymphoma (PTCL) is a heterogeneous disease that accounts for 5%-10% of all non-Hodgkin lymphomas (NHL) in Western countries and 15-20% of all lymphomas in Asia [1, 2]. Nodal PTCL is the common subtype of PTCL, including PTCL not otherwise specified (PTCL-NOS, 25%), angioimmunoblastic T-cell lymphoma (AITL, 18%), anaplastic large-cell lymphoma (ALCL), and both ALK positive (6%) and ALK negative (5%) [1, 3]. Cyclophosphamide, doxorubicin, vincristine, prednisone (CHOP), or CHOP-like regimens have been the most commonly used treatment strategies for nodal PTCL [4, 5]. Most PTCL patients have poor prognosis, with a 5-year overall survival (OS) between 32% and 49% [1, 6]. In the past decade, the International Prognostic Index (IPI) and National Comprehensive Cancer Network International Prognostic Index (NCCN-IPI) are the most widely used prognostic indicators for patients with aggressive lymphoma [1, 7–9]. However, they cannot easily identify this high-risk population [10, 11]. Therefore, reliable prognostic factors are needed to better identify populations at high risk.

^18^F-fluorodeoxyglucose positron emission tomography/computed tomography (^18^F-FDG PET/CT) is now recommended for clinical staging and initial assessment of PTCL [12]. Several studies have confirmed that total metabolic tumor volume (TMTV) and total lesion glycolysis (TLG) obtained from baseline PET/CT are associated with the prognosis of Hodgkin's lymphoma (HL), follicular lymphoma (FL), diffuse large B-cell lymphoma (DLBCL), and extranodal natural killer/T-cell lymphoma (ENKTL) [13–17]. However, the prognostic value of ^18^F-FDG PET/CT quantitative parameters (TMTV and TLG) in PTCL patients remains largely unclear. In the present study, we aimed to investigate whether the metabolic parameters TMTV and TLG could be used to predict prognosis in PTCL.

## 2. Materials and Methods

### 2.1. Patients

A total of 51 PTCL patients who underwent pre-treatment ^18^F-FDG PET/CT from March 2013 to May 2019 were enrolled in the present study. Inclusion criteria were set as follows: (1) histopathologically confirmed as PTCL (PTCL-NOS, AITL, or ALCL ALK-) and (2) availability of digital image data for analysis. ALCL ALK+ patients who had superior outcome after CHOP or CHOP-like regimens were excluded.

Characteristics of patients included age, gender, B symptoms, LDH (lactate dehydrogenase) level, IPI score, NCCN-IPI score, prognostic index for T-cell lymphoma (PIT) score, Eastern Cooperative Oncology Group (ECOG) performance status, Ann Arbor stage, bone marrow biopsy, and PET/CT data.

### 2.2. PET/CT Acquisition

All patients underwent ^18^F-FDG PET/CT images (Discovery STE; General Electric Medical Systems, Milwaukee, WI, USA). Patients were fasted for at least 6 h before the ^18^F-FDG PET/CT, and the blood glucose level was lower than 11 mmol/L. PET and CT images were obtained at 60 ± 10 min after the tracer injection (4.07–5.55 MBq/kg). CT images were acquired at 120 mA, 140 kV, transaxial FOV of 70 cm, pitch of 1.75, rotation time of 0.8 s, and slice thickness of 3.75 mm. PET emission images were acquired from the top of the skull to the upper thigh, 2 min per bed position. PET images were reconstructed with iterative algorithms, with CT data for attenuation correction.

### 2.3. Image Analysis

All images were retrospectively analyzed using Advantage Workstation 4.3_05 by two experienced nuclear medicine physicians. Maximal standardized uptake value (SUV_max_) was determined as the highest SUV of the pixel in the region of interest (ROI). Baseline TMTV, summing the volumes of all hyper-metabolic lesions, was computed using the SUV_max_ threshold of 41% [18]. Bone marrow involvement was considered in volume measurement only if there was focal uptake. Spleen was considered as involved if there was focal uptake or diffuse uptake higher than 150% of the liver background [19]. The TLG was calculated using the following equation: TLG = MTV∗SUV_mean_.

### 2.4. Statistical Analysis

Statistical analyses were performed using GraphPad Prism 5.0 software (San Diego, CA, USA) and SPSS 22.0 software (IBM, Chicago, IL, USA). Differences in clinical variables between TMTV and TLG groups were analyzed by Pearson chi-squared test and Fisher's exact test. Correlation between TMTV or TLG and clinical prognostic factors was assessed using the Spearman's rank correlation test. Receiver-operating characteristic (ROC) analysis was used to determine the optimal cutoff values for SUV_max_, TMTV, and TLG. Progression-free survival (PFS) was defined as the time from diagnosis until lymphoma progression, death from any cause, or last follow-up. OS was defined as the time from diagnosis until death from any cause or last follow-up [20]. Survival curves were calculated by Kaplan-Meier analysis, and comparisons between the groups were made using a log-rank test. Cox proportional hazards model was used for multivariate survival analysis. A *p* < 0.05 was considered as statistically significant.

## 3. Results

### 3.1. Characteristics of Patients

A total of 51 PTCL patients who underwent pre-treatment ^18^F-FDG PET/CT were retrospectively enrolled in this study. Our data showed that 38 patients had PTCL-NOS, eight patients had AITL, and five patients were ALCL ALK-. Their median age was 56 years (range, 15-88 years). Moreover, 34 (84.31%) patients received R-CHOP or CHOP-like (CHOEP, miniCHOP) regimes, of which nine patients received autologous stem cell transplantation and three patients received allogeneic stem cell transplantation. The remaining eight (15.69%) patients received other therapeutic regimens. The median follow-up time was 18 months (range, 2-82 months). In addition, 22 patients had disease progression with a median time of 8 months (range, 1–22 months), and 18 patients died with a median time of 9 months (range, 2–24 months). The 2-year PFS and OS were 56.86% and 64.71%, respectively.  Table 1 summarizes the patient's characteristics.

### 3.2. Relationship between Clinical Factors and Metabolic Parameters

The median value of TLG, TMTV, and SUV_max_ was 296.464 (4.8-6497.28), 62.880 cm^3^ (3.81-1485.38 cm^3^), and 8.48 (1.27-32.65), respectively. The optimal cutoff values of TLG, TMTV, and SUV_max_ obtained using the ROC curve were 270.725 (sensitivity 88.9%, specificity 66.7%, AUC 0.749, *p* = 0.004), 62.405 cm^3^ (sensitivity 77.8%, specificity 63.6%, AUC 0.702, *p* = 0.018), and 9.545 (sensitivity 55.6%, specificity 75.8%, AUC 0.644, *p* = 0.092), respectively.

Table 2 shows the relationship between clinical characteristics and metabolic parameters. High TMTV (>62.405 cm^3^) and TLG (>270.725) were associated with stage III/IV (*p* < 0.001 and *p* = 0.020, respectively), greater extranodal involvement (*p* = 0.032 and *p* = 0.023, respectively), and higher IPI scores (*p* = 0.007 and *p* = 0.002, respectively). Meanwhile, high TMTV was also associated with poor performance status (*p* = 0.048), and high TLG was also associated with high SUV_max_ (*p* < 0.001).

### 3.3. Role of ^18^F-FDG PET/CT in Outcome Prediction

**Figure 1:**
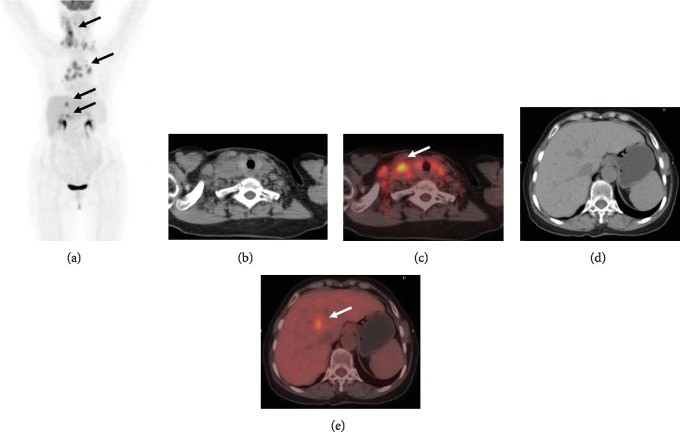
A 78-year-old woman was diagnosed with PTCL-NOS. The baseline PET/CT image showed increased ^18^F-FDG uptake in the cervical, mediastinum, and abdominal lymph nodes and liver with high TLG (357.518) and TMTV (90.76 cm^3^). The patient died 10 months after follow-up.

**Figure 2:**
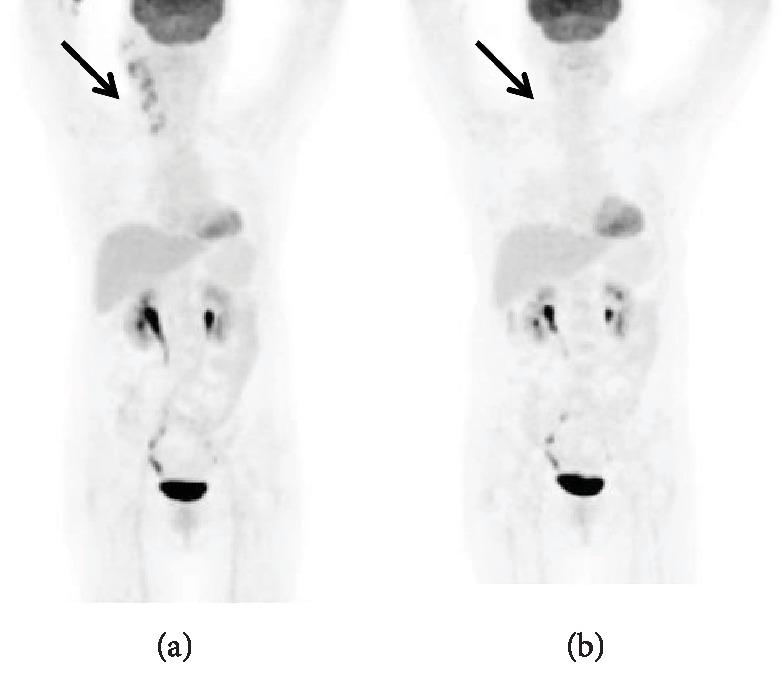
A 59-year-old female was diagnosed with PTCL-NOS. The baseline PET/CT image showed increased ^18^F-FDG uptake in the right cervical lymph node with low TLG (102.219) and TMTV (26.2 cm^3^). The last PET/CT after six cycles of R-CHOP therapy did not show hypermetabolic lesions. The patient was still alive after 50 months of follow-up.

**Figure 3:**
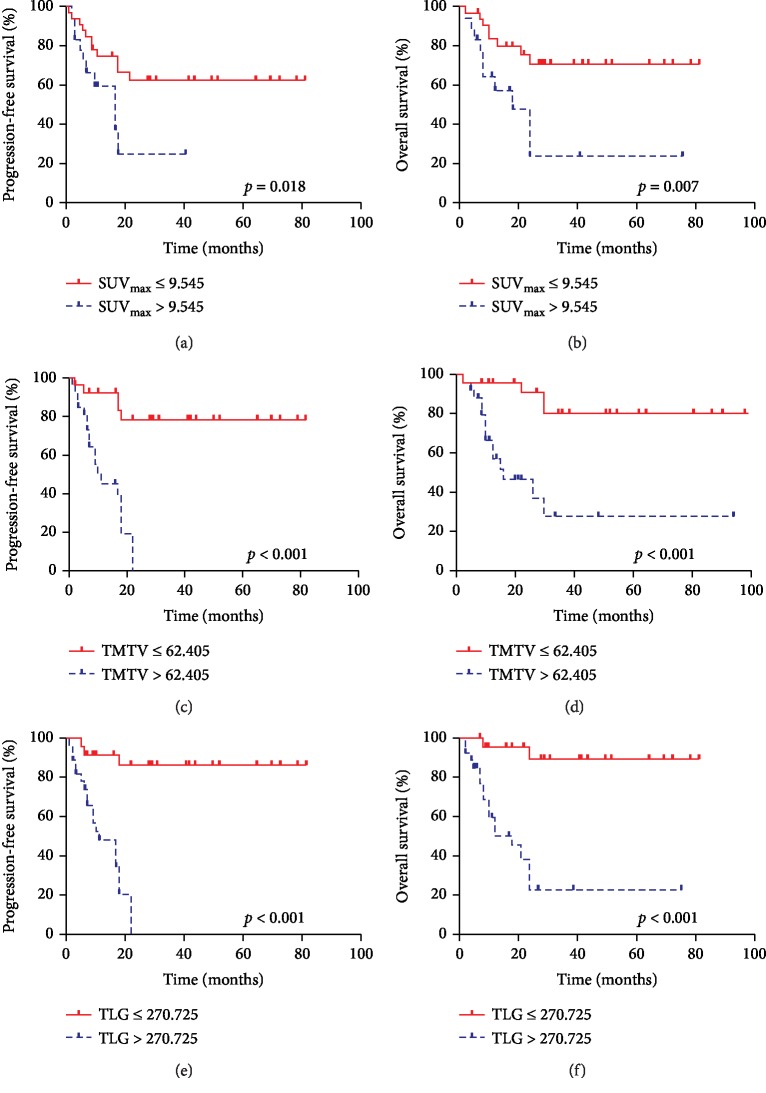
Kaplan-Meier survival analysis of PFS and OS in PTCL patient according to (a, b) SUV_max_, (c, d) TMTV, and (e, f) TLG.

Kaplan-Meier analysis revealed that patients with low TLG, TMTV, and SUV_max_ levels had a better clinical outcome than those with high TLG, TMTV, and SUV_max_ levels (Figures 1–3). The 2-year PFS rate of the high- and low-TMTV groups was 34.62% and 80%, respectively (*p* < 0.001). The 2-year OS rate of the high- and low-TMTV groups was 46.15% and 84.00%, respectively (*p* < 0.001). The median OS was 13 months in patients with higher TMTV (>62.405). The 2-year PFS rate of the high- and low-TLG groups was 29.63% and 87.50%, respectively (*p* < 0.001). The 2-year OS rate of the high- and low-TLG groups was 40.74% and 91.67%, respectively (*p* < 0.001). The median OS was 18 months in patients with higher TLG (>270.725).

In univariate analysis (Table 3), ECOG status, IPI scores, TLG, TMTV, and SUV_max_ were all correlated with both PFS and OS whereas age, NCCN-IPI, and PIT scores were correlated with only OS but not PFS. By Spearman's rank correlation test, there was a strong correlation between TMTV and TLG (*r* = 0.929, *p* < 0.001, Table 4). Therefore, TMTV or TLG was, respectively, incorporated into a multivariate analysis with other clinical features. In multivariate analysis (Table 5), TLG and TMTV were independent prognostic factors of both PFS (HR 11.562, 95% CI 3.218-41.542, *p* < 0.001 and HR 7.061, 95% CI 2.464-20.229, *p* < 0.001, respectively) and OS (HR 11.609, 95% CI 2.595-51.930, *p* = 0.001 and HR 5.026, 95% CI 1.538-16.421, *p* = 0.008, respectively). However, when TMTV was incorporated in multivariate analysis, SUV_max_ and NCCN-IPI were also independent predictors of OS (HR 3.161, 95% CI 1.197-8.346, *p* = 0.020 and HR 3.112, 95% CI 1.109-8.732, *p* = 0.031, respectively) and SUV_max_ showed a trend as an independent predictor of PFS (*p* = 0.096).

### 3.4. Combination of TMTV and NCCN-IPI Scores

**Figure 4:**
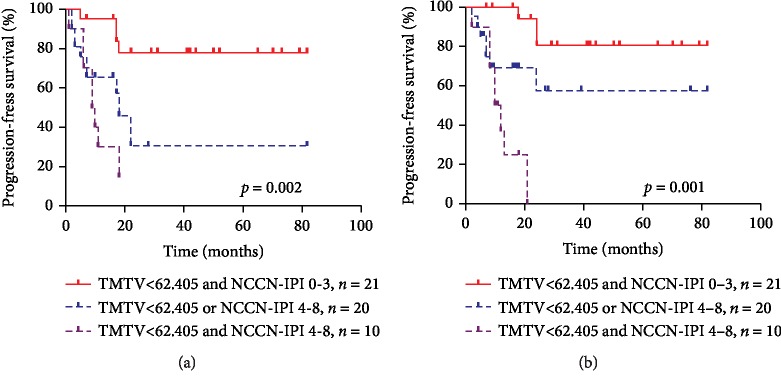
Kaplan-Meier survival analysis of PFS and OS in PTCL patient according to the TMTV and NCCN-IPI scores (a, b).

Combination of TMTV and NCCN-IPI scores gave an added predictive value, patients were divided into three risk groups as follows: low-risk group, TMTV ≤ 62.405 cm^3^ and NCCN-IPI score of 0-3 (*n* = 20); intermediate-risk group, TMTV > 62.405 and NCCN-IPI score of 0-3 or TMTV ≤ 62.405 and NCCN-IPI score of 4-8 (*n* = 21); and high-risk group, TMTV > 62.405 cm^3^ and NCCN-IPI score of 4-8 (*n* = 10). The 2-year PFS of these three groups was 80.00%, 52.40%, and 20.00%, respectively, and the 2-year OS of the above-mentioned three groups was 85.00%, 66.70%, and 20.00%, respectively. These groups had significantly different PFS (*χ*^2^ = 14.307, *p* = 0.002; Figure 4(a)) and OS ( *χ*^2^ = 17.851, *p* < 0.001; Figure 4(b)). In a subanalysis, we found that the PFS and OS of patients in the low-risk group were significantly better compared with the intermediate-risk group (*χ*^2^ = 6.929, *p* = 0.008 and  *χ*^2^ = 4.053, *p* = 0.044, respectively) and high-risk group (*χ*^2^ = 14.569, *p* < 0.001 and *χ*^2^ = 24.546, *p* < 0.001, respectively). Moreover, there were differences in PFS and OS between the intermediate-risk and high-risk groups although such differences were not significant (*χ*^2^ = 1.793, *p* = 0.181 and *χ*^2^ = 3.839, *p* = 0.050, respectively).

## 4. Discussion

The International Conference on Malignant Lymphoma (ICML) recommends investigating the quantitative parameters of ^18^F-FDG PET/CT for prognostic analysis [21]. Several studies have demonstrated that tumor burden is a poor prognostic factor for different subtypes of lymphoma [13–17, 22]. This has led to an increased interest in assessing prognosis using baseline TMTV and TLG, occasionally in combination with clinical scores [23, 24].

In this retrospective study, we investigated the prognostic value of TMTV and TLG at baseline PET/CT and found that patients with high TMTV or TLG values showed shorter PFS and OS than those with low TMTV and TLG values. Mehta-Shah et al. [19] has reported that baseline TMTV is an independent predictor of PFS and OS in PTCL patients. Cottereau et al. [25] have conducted a multicenter retrospective analysis on 140 nodal PTCL patients and confirmed a poor prognostic value for the high TMTV in baseline PET/CT images for the prediction of PFS and OS. These results are similar to ours. However, we also confirmed that both baseline TMTV and TLG, which were not included in other reports, were also independent prognostic factors of PFS and OS in nodal PTCL in our study. Nevertheless, some studies have different results [23, 26]. Cottereau et al. [23] have found that the TMTV rather than TLG remains the only independent predictor for both PFS and OS in PTCL patients and high values of TMTV predict a worse prognosis. In a multicenter retrospective study, Pak et al. [26] have found that baseline TLG is the only independent prognostic factor for PFS in patients with extranodal nasal-type NK/T cell lymphoma. We speculated that such discrepancy could be attributed to the strong correlation between TMTV and TLG, leading to a wrong assessment when they are all included in the multivariate analysis.

Furthermore, NCCN-IPI score was also an independent prognostic factor for OS. Patients with an NCCN-IPI score of 0-3 had better clinical outcome than the group with an NCCN-IPI score of 4-8. Some studies have shown that combination of baseline PET/CT parameters and clinical prognostic indices allows us to stratify the progression risk of lymphoma patients [23, 24, 27, 28]. In our present study, we combined TMTV > 62.405 cm^3^ and NCCN-IPI score to stratify patients into three risk categories. Patients with both TMTV > 62.405 cm^3^ and an NCCN-IPI score of 4-8 had a very poor outcome, with a median OS of 10 months.

SUV_max_ is the most widely used indicator in clinical practice. In a retrospective study consisting of 86 patients, Hwang et al. [29] have shown that patients with a higher SUV_max_ value show worse prognosis. Pak et al. [26] and Chang et al. [30] have shown similar results that a higher SUV_max_ value is significantly associated with tumor aggressiveness in patients with T-cell lymphoma. However, there are also controversial results. Some studies have suggested that there is no significant correlation between SUV_max_ and prognosis in patients with aggressive NHL [31–33]. In our present study, we demonstrated that SUV_max_ was an independent predictor of OS but not PFS. Such difference could be attributed to the heterogeneity of different lymphomas. In addition, the SUV_max_ only represents the glucose metabolism of the most aggressive tumor tissue, which might be another reason for the different outcomes, especially in aggressive NHL.

In some studies, the absolute threshold of SUV ≥2.5 is used to calculate MTV [15, 34]. However, SUV values are likely to be affected by partial volume effect, time after injection, and blood glucose level [35, 36]. In our present study, MTV was measured using a SUV_max_ threshold of 41% [18]. This potentially overestimated the lesion volume of small tumors. However, only one patient had the volume of tumor < 4 cm^3^ in our study. In addition, this also potentially underestimated the lesion volume of high SUV_max_. However, in our study, only four patients had SUV_max_ > 15 and there was no significant difference in SUV_max_ between patients with higher or lower TMTV. The 41% SUV_max_ threshold method shows an excellent interobserver agreement, and it has been used in different subtypes of lymphoma [14, 27, 37–39]. To the best of our knowledge, there is no consensus on the MTV calculation method. Recent studies have shown that baseline TMTV values are significantly affected by the choice of the marginal threshold methods [40]. Therefore, it is necessary to define the metabolic volume using an accurate and standardized method.

This study has some limitations. First, this was a single-center retrospective analysis with a relatively short follow-up. In particular, four patients were followed for no more than 6 months because they died of progressive PTCL. Additionally, the number of patients who underwent ^18^F-FDG PET/CT after 3–4 cycles of chemotherapy (*n* = 18) and after all planned first-line therapy (*n* = 25) was quite small. Therefore, the prognostic role of interim and end-of-treatment PET/CT should be further validated in future trials consisting of larger patient samples.

## 5. Conclusions

Baseline TMTV and TLG were independent predictors of PFS and OS in PTCL patients, and SUV_max_ and NCCN-IPI scores were also independent predictors of OS. Moreover, the combination of TMTV and NCCN-IPI scores improved patient risk-stratification at the initial stage, which might contribute to the adjustment of the therapeutic regime.

## Figures and Tables

**Table 1:** Characteristics of patients.

Characteristic	No. of patients (*n* = 51)
Sex (male)	35 (68.63%)
Age median (range)	56 (15-88)
B symptoms (yes)	28 (54.90%)
Ann Arbor stage (III/IV)	41 (80.39%)
ECOG > 1	7 (13.73%)
BM (+)	7 (13.73%)
LDH (increased)	24 (47.06%)
IPI	
0-1	18 (35.29%)
2	11 (21.57%)
3	14 (27.45%)
4-5	8 (15.69%)
NCCN-IPI	
0-3	36 (70.59%)
4-8	15 (29.41%)
No. of extranodal sites ≥ 2	22 (43.14%)
PIT	
0	16 (31.37%)
1	21 (41.18%)
2	10 (19.61%)
3-4	4 (7.84%)
TMTV mean (range)	62.880 (3.81-1485.38)
TLG mean (range)	296.464 (4.8-6497.28)
SUV_max_ mean (range)	8.48 (1.27-32.65)
Subtype	
PTCL-NOS	38 (74.51%)
AITL	8 (15.69%)
ALK-ALCL	5 (9.80%)

Abbreviations: ECOG: Eastern Cooperative Oncology Group; BM: bone marrow; IPI: International Prognostic Index; NCCN-IPI: National Comprehensive Cancer Network International Prognostic Index; PIT: prognostic index for T-cell lymphoma.

**Table 2:** Comparison between low and high TMTV and TLG groups.

	TMTV			TLG		
	High (*N* = 26)	Low (*N* = 25)	*p*	High (*N* = 27)	Low (*N* = 24)	*p*
Sex						
Female	11	5	0.086	11	5	0.126
Male	15	20		16	19	
Age						
>60	10	8	0.629	11	7	0.388
≤60	16	17		16	17	
B symptoms						
Yes	17	11	0.125	17	11	0.220
No	9	14		10	13	
Ann Arbor stage						
I-II	0	10	<0.001^∗^	2	8	0.020^∗^
III/IV	26	15		25	16	
ECOG						
>1	6	1	0.048^∗^	6	1	0.061
≤1	20	24		21	23	
BM						
Yes	4	3	0.725	4	3	0.811
No	22	22		23	21	
LDH						
Increased	15	9	0.121	15	9	0.197
—	11	16		12	15	
NCCN-IPI						
0-3	16	20	0.148	16	20	0.060
4-8	10	5		11	4	
No. of extranodal sites						
≥2	15	7	0.032^∗^	16	6	0.023^∗^
<2	11	18		11	18	
IPI						
≥3	16	6	0.007^∗^	17	5	0.002^∗^
<3	10	19		10	19	
PIT						
>1	10	4	0.072	10	4	0.104
≤1	16	21		17	20	
SUV_max_						
>9.545	12	6	0.098	16	2	<0001^∗^
≤9.545	14	19		11	22	

Note: ^∗^Statistically significant. Abbreviations: ECOG: Eastern Cooperative Oncology Group; BM: bone marrow; IPI: International Prognostic Index; NCCN-IPI: National Comprehensive Cancer Network International Prognostic Index; PIT: prognostic index for T-cell lymphoma.

**Table 3:** Univariate analysis for survivals.

	PFS			OS		
	HR	95% CI	*p*	HR	95% CI	*p*
Sex (male)	0.766	0.305-1.927	0.571	2.232	0.787-6.333	0.131
Age > 60	2.124	0.861-5.239	0.102	2.894	1.056-7.932	0.039^∗^
B symptoms	1.837	0.781-4.321	0.164	1.501	0.586-3.848	0.398
Ann Arbor stage (III/IV)	1.430	0.529-3.866	0.381	1.685	0.574-4.946	0.342
ECOG > 1	5.536	1.259-24.350	0.024^∗^	10.660	2.063-55.020	0.005^∗^
BM involvement	0.917	0.274-3.070	0.889	1.337	0.337-5.310	0.680
LDH	1.261	0.535-2.973	0.595	1.757	0.677-4.561	0.247
IPI ≥ 3	3.206	1.296-7.929	0.012^∗^	4.954	1.801-13.630	0.002^∗^
NCCN-IPI 4-8	2.370	0.886-6.339	0.086	4.610	1.507-14.100	0.007^∗^
PIT > 1	2.032	0.747-5.524	0.165	3.615	1.167-11.200	0.026^∗^
No. of extranodal sites ≥ 2	2.128	0.881-5.140	0.093	1.584	0.604-4.149	0.350
TMTV > 62.405	7.004	2.802-17.510	<0.001^∗^	6.467	2.387-17.520	<0.001^∗^
TLG > 270.725	8.233	3.370-20.110	<0.001^∗^	8.365	3.163-22.120	<0.001^∗^
SUV_max_ > 9.545	3.193	1.220-8.353	0.018^∗^	4.278	1.488-12.300	0.007^∗^

Note: ^∗^Statistically significant. Abbreviations: ECOG: Eastern Cooperative Oncology Group; BM: bone marrow; IPI: International Prognostic Index; NCCN-IPI: National Comprehensive Cancer Network International Prognostic Index; PIT: prognostic index for T-cell lymphoma.

**Table 4:** Correlation between clinical characteristics with semiquantitative parameters.

	TMTV		TLG	
	*r*	*p*	*r*	*p*
Sex	−0.169	0.235	−0.118	0.411
Age	0.072	0.613	0.059	0.683
B symptoms	0.169	0.237	0.185	0.194
Ann Arbor stage	0.436	0.001^∗^	0.379	0.006^∗^
ECOG	0.314	0.025^∗^	0.321	0.022^∗^
No. of extranodal sites	0.433	0.002^∗^	0.414	0.003^∗^
BM involvement	0.135	0.343	0.066	0.646
LDH	0.240	0.090^∗^	0.201	0.157
IPI	0.538	<0.001^∗^	0.481	<0.001^∗^
NCCN-IPI	0.327	0.019^∗^	0.298	0.034^∗^
PIT	0.343	0.014^∗^	0.270	0.055
SUV_max_	0.320	0.022^∗^	0.597	<0.001^∗^
TMTV	—	—	0.929	<0.001^∗^
TLG	0.929	<0.001^∗^	—	—

Note: ^∗^Statistically significant. Abbreviations: ECOG: Eastern Cooperative Oncology Group; BM: bone marrow; IPI: International Prognostic Index; NCCN-IPI: National Comprehensive Cancer Network International Prognostic Index; PIT: prognostic index for T-cell lymphoma.

**Table 5:** Multivariate analysis for survivals.

	PFS				OS		
	HR	95% CI	*p*		HR	95% CI	*p*
TMTV							
TMTV	7.061	2.464-20.229	<0.001^∗^	TMTV	5.026	1.538-16.421	0.008^∗^
SUV_max_	—	—	0.096	SUV_max_	3.161	1.197-8.346	0.020^∗^
ECOG > 1	—	—	0.229	NCCN-IPI	3.112	1.109-8.732	0.031^∗^
IPI	—	—	0.233	ECOG > 1	—	—	0.499
NCCN-IPI	—	—	0.515	IPI	—	—	0.856
				PIT	—	—	0.409
				Age	—	—	0.299
TLG							
TLG	11.562	3.218-41.542	<0.001^∗^	TLG	11.609	2.595-51.930	0.001^∗^
SUV_max_	—	—	0.794	SUV_max_	—	—	0.360
ECOG > 1	—	—	0.206	ECOG > 1	—	—	0.052
IPI	—	—	0.398	NCCN-IPI	—	—	0.325
NCCN-IPI	—	—	0.794	IPI	—	—	0.216
				PIT	—	—	0.703
				Age	—	—	0.170

Note: ^∗^Statistically significant. Abbreviations: ECOG: Eastern Cooperative Oncology Group; BM: bone marrow; IPI: International Prognostic Index; NCCN-IPI: National Comprehensive Cancer Network International Prognostic Index; PIT: prognostic index for T-cell lymphoma.

## Data Availability

The data used to support the findings of this study are available from the corresponding author upon request.
